# Determining the association between hypertension and bone metabolism markers in osteoporotic patients

**DOI:** 10.1097/MD.0000000000026276

**Published:** 2021-06-18

**Authors:** Zhuoqing Hu, Kevin Yang, Zhihui Hu, Miaosheng Li, Hao Wei, Zheng Tang, Baitong Chen, Chengbiao Su, De Cai, Jinrong Xu

**Affiliations:** a Department of Cardiovascular Internal Medicine, The Second Affiliated Hospital of Guangdong Medical University, Zhanjiang; b Department of Endocrinology, The Second Affiliated Hospital of Guangzhou Medical University; c Dept of Cardiology, Sun Yat-sen University, Guangzhou; d Guangdong Medical University; e Department of Pharmacy, Affiliated Hospital of Guangdong Medical University, Zhanjiang, China.

**Keywords:** bone metabolism markers, hypertension, osteoporosis

## Abstract

The aim of the case study is to examine the association between hypertension and the level of bone metabolism markers in newly diagnosed osteoporotic patients.

A cross-sectional study of 518 subjects was done to see the association between hypertension and the level of osteocalcin (OC), bone-specific alkaline phosphatase (B-ALP), Tartrate-resistant acid phosphatase (TRAP.5B), and 25-hydroxy vitamin D (25-OHD). There were 243 (46.9%) osteoporosis patients with hypertension. Both univariate and multivariate analysis have suggested that lower OC and 25-OHD levels were associated with hypertension. The potential confounders-adjusted OC level was significantly lower in hypertensive female group than that in the female without hypertension group [β = -0.20, 95% confidence interval (95% CI) = -0.37 to -0.03, *P* = .02 in final adjust model]. The potential confounders-adjusted 25-OHD level was significantly lower in hypertensive male group than that in male without hypertension group (β = -0.34, 95% CI = -0.58 to -0.10, *P* = .01 in final adjust model). The B-ALP and TRACP.5B levels were positively associated with hypertension in all patients or subgroup analysis. However, all the correlations had no statistical significance for the B-ALP and TRACP.5B.

In conclusion, the hypertension was associated with low level of OC and 25-OHD. Hypertension probably led to low bone turnover, which may be one of the mechanisms of hypertension-related osteoporosis.

## 1 Introduction

Osteoporosis is a metabolic bone disease, which is characterized by decrease of bone mass as well as degeneration of bone microstructure.^[1]^ Also, the incidence of osteoporosis has a direct relationship with the age of the patient. From the last decade, the prevalence of osteoporosis has increased from 14.94% before to 27.96%.^[2]^ During the recent decades, it was reported that the prevalence of Chinese adults with hypertension had increased significantly to 27.8% with hypertension.^[3]^ Many studies evaluating a relationship between hypertension and osteoporosis have been published in the past and there were significant evidences indicating that high blood pressure is associated with increased bone loss.^[4–6]^ Furthermore, the association between high blood pressure and bone loss may have contributed to the risk of fractures.^[4,7]^

Hypertension is thought to be linked to bone health through chronic elevation in the levels of parathyroid hormone (PTH), angiotensin II, and catecholamines including adrenaline. In addition, hypertensive patients have decreased intestinal absorption, increased urinary calcium excretion, and decreased plasma vitamin D concentrations, which promote PTH continuous secretion.^[8]^ Also, sustained elevation of PTH contribute to bone resorption by increasing osteoclast differentiation.^[9]^ Activation of the renin-angiotensin system in hypertensive mice accelerates bone resorption, induces high bone turnover osteoporosis,^[10]^ and it may eventually increase the risk of fragile fractures.

Although the influence of hypertension has been elaborated in animal model, there are little amount of clinical data indicating whether hypertension independently influences bone turnover. Osteocalcin (OC) and bone-specific alkaline phosphatase (B-ALP) were commonly used to evaluate bone formation and 25-OHD was considered as bone mineralization regulator. In addition, TRACP has properties indicating that it can be a good marker of bone resorption and osteoclast activity. Finding the amount of TRACP in the serum, especially TRACP-5b, can help understand bone metabolism under physiological conditions and pathological conditions. Previous studies on the relationship between blood pressure and bone formation have demonstrated conflicting conclusions, which is that OC was shown inversely or positively related to high blood pressure.^[11–13]^ Higher blood pressure was also significantly correlated with increased ALP, but there were few studies showing the correlation between B-ALP and hypertension. There was also a consistent result of which the hypertensive patients had vitamin D insufficiency, which was involved in the pathogenesis of bone loss, but there was no consistent result showing the relationship between TRACP.5B and hypertension.^[14]^ In order to further elucidate the role of hypertension in bone metabolism, the purpose of this study is to examine the association between hypertension and bone metabolism markers in osteoporotic patients.

## 2 Methods

### 2.1 Study participants

All patients were newly diagnosed with osteoporosis were admitted to orthopedics department of the Affiliated Hospital of Guangdong Medical University in China from January 2013 to January 2016. A total of 518 patients with osteoporosis were enrolled in this study after rigorous diagnosis and exclusion criteria. The project was performed in accordance with the principles of the Declaration of Helsinki and it is approved through the Ethics Committee of the Affiliated Hospital of Guangdong Medical University. We obtained written informed consent from all participants.

### 2.2 Diagnosis and exclusion criteria

All the patients were diagnosed with osteoporosis and admitted to our hospital for the first time. In short, each eligible patient met the following inclusion criteria: his or her diagnosis was primary osteoporosis which was confirmed and signed by the chief physician and attending physician; age ≥50 years. Subjects were excluded if they were below 50 years; were the premenopausal female patients; no sure that patients newly diagnosed with osteoporosis; were caused by other causes such as accident, trauma, tumor; were the patients who had a history of fragile fracture; had severe cardiovascular and cerebrovascular diseases, severe liver and kidney dysfunction, severe infection; and receiving glucocorticoids or Vitamin D treatment within 4 months.

The case study was hospital-based cross-sectional study and the clinical data were extracted from the medical records. Information including the age, gender, occupation, fragile fracture, related medical history, and anti-hypertensive medication history were collected for each patient. Osteoporosis was diagnosed from the participants in accordance to the criteria issued from World Health Organization, which is that the T-score ≤2.5 in the regions of lumbar spine, femur neck, or hip by bone mineral density testing.^[15]^ In addition, the diagnostic criteria for hypertension was based on the World Health Organization, which is that the findings of systolic blood pressure and diastolic blood pressure must be greater ≥140 and ≥90 mm Hg, respectively.^[16]^

During the morning of the second day of admission, all the selected patients were on fasting condition when the venous blood was extracted from them. The blood was placed in the vacuum blood collection tube without coagulants or anticoagulants. They were then stored in the refrigerator at 4°C for more than 1 hour. Afterwards, the blood was taken out and was let sat out for 30 minutes at 25° C to allow the blood to coagulate. Next, the blood was centrifuged at 2000 × g for 15 minutes at 4°C for the serum to be separated from the blood. In the serum, biochemical indicators were detected which include B-ALP, OC, TRACP, and 25-OHD. Roche Cobas E601 (Chemiluminescence method) and supporting kits were used to detect the serum biochemical indicators of B-ALP, OC, TRACP, and 25-OHD. ARCHITECT c16000 (Arsenazo III method) and calcium determination kit were used for the quantitative analysis of calcium in serum. All the biochemical examinations were completed in the clinical lab of the Affiliated Hospital of Guangdong Medical University. Normal bone metabolism markers levels ranges are summarized in Table 1. Furthermore, all clinical records of the eligible cases were manually proofread by another researcher in a blinded fashion.

**Table 1:** Characteristics of the osteoporotic patients.

Characteristic	Without-hypertension	With-hypertension	*P*
Age, yr	73.59 ± 10.72	77.26 ± 9.16	<.001
Sex (women, %)	202 (73.45%)	168 (69.14%)	.27
BMI, kg/m^2^	22.78 ± 4.60	23.12 ± 4.54	.40
SBP	105 ± 15	139 ± 13	<.001
DBP	78 ± 9	98 ± 10	<.001
Occupation			.002
Light physical labor	90 (32.73%)	104 (42.80%)	
Moderate physical labor	83 (30.18%)	83 (34.16%)	
Hard physical labor	102 (37.09%)	56 (23.05%)	
Total Ca, mmol/L^∗^	2.20 (1.50–3.40)	2.19 (1.86–3.60)	.79
OC, ng/mL^∗^	19.86 (1.93–109.40)	17.51 (2.72–64.86)	.02
25-OHD, ng/mL^∗^	22.10 (2.73–70.00)	20.48 (3.00–55.34)	.10
B-ALP, μg/L^∗^	0.56 (0.10–2.43)	0.58 (0.10–1.39)	.51
TRACP.5B, mlU/mL^∗^	39.87 (3.80–274.60)	42.21 (9.42–159.1)	.72
Comorbidities			
Type 2 diabetes	24 (8.73%)	35 (14.40%)	.04
Cerebral infarction	9 (3.27%)	19 (7.82%)	.02
Coronary heart disease	16 (5.82%)	34 (13.99%)	.002
Disease of respiratory system	20 (7.27%)	12 (4.94%)	.21
Osteoarthritis	25 (9.09%)	26 (10.70%)	.54
Chronic renal insufficiency	5 (1.82%)	6 (2.47%)	.61
Anti-hypertension drugs used			<.001
No	274 (99.64%)	192 (79.01%)	
Yes	1 (0.36%)	51 (20.99%)	

### 2.3 Statistical analysis

Empower(R) (www.empowerstats.com, X&Y solutions, Inc Boston, MA) and R (http://www.R-project.org) were applied to all statistical analyses in the research. Data were presented as mean ± SD, proportions, or median (range). Subjects were divided into non-hypertensive and hypertensive groups. The relationship between hypertension and bone metabolism markers were assessed in both univariate and multivariate linear regression analysis. The potential covariates were screened by being adjusted through generalized linear models. The screening criteria included risk factors producing >10% change in the regression coefficient after introduction into the basic model. In order to normalize the data distribution, the Log of bone markers was used for calculation. All *P*-value of less than .05 (2-tailed) were defined as statistical significance.

## 3 Results

### 3.1 Clinical characteristics of study groups

The baseline characteristics of the study participants are presented in Table 1. The mean age of the total subjects was 75.15 ± 10.24 years. 71.11% of the participants were females. There were 243 (46.9%) osteoporosis patients with hypertension. Diseases of respiratory system among the participants included chronic obstructive pulmonary disease, chronic bronchitis.

### 3.2 Univariate regression for the relationship between hypertension and bone metabolism markers

Table 2 showed the univariate regression for the relationship between hypertension and 4 variables, which include the OC log2 transform, B-ALP log2 transform, 25-OHD log2 transform, and TRAP.5B log2 transform. In this univariate analysis, there were no variables being adjusted. In the hypertensive groups, the level of OC was 0.12 units lower than the no hypertensive group [95% confidence interval (95% CI): -0.26 to 0.02, *P* = .09]. The level of B-ALP in the hypertensive group increased by 0.07 Unit (95% CI: -0.26 to 0.40, *P* = .68). As for the TRAP.5B in the hypertensive group, the level was increased by 0.03 Unit (95% CI: -0.29 to 0.36, *P* = .84). For 25-OHD level in the hypertensive group, the level was decreased by 0.11 (95% CI: -0.24 to 0.01, *P* = .06).

**Table 2:** Univariate regression for the relationship between hypertension and bone metabolism markers (β, 95% CI, *P* value).

	OC	ALP	25-OHD	TRACP.5B
Without-hypertension	Reference	Reference	Reference	Reference
With-hypertension	-0.12 (-0.26, 0.02) 0.09	0.07 (-0.26, 0.40) 0.68	-0.11 (-0.24, 0.01) 0.06	0.03 (-0.29, 0.36) 0.84

### 3.3 Multivariate regression for the relationship between hypertension and bone metabolism markers

Table 3 detailed univariate and multivariate regressions for the relationship between hypertension and bone metabolism markers under Log2-median transformation. Adjust I model showed that the level of OC was lower by 0.2 unit for hypertensive females (95% CI: -0.39 to -0.07, *P* = .004), and 0.16 unit for total subjects (95% CI: -0.30 to -0.02, *P* = .02) compared with the non-hypertensive females. After adjusting for more potential bias variables in adjusted II model, the result of multivariate analysis suggested that OC level was significantly lower in female subject with hypertension (β= -0.2, 95% CI: -0.37 to -0.03, *P* = .02) compared with the non-hypertensive females. However, the correlation in the total subjects had no statistical significance (β= -0.11, 95% CI: -0.26 to 0.03, *P* = .13). In adjust I model and Adjust II model, men in the hypertensive group showed higher serum OC level than those in the non-hypertensive group, but there was no statistical significance.

**Table 3:** Multivariate regression for the relationship between hypertension and the bone metabolism markers (β, 95% CI, *P* value).

		Men	Women	Total
Adjust I	Without-hypertension	Reference	Reference	Reference
	With-hypertension			
	OC	0.00 (-0.28, 0.29) 0.98	-0.23 (-0.39, -0.07) 0.004	-0.16 (-0.30, -0.02) 0.02
	B-ALP	0.46 (-0.23, 1.14) 0.20	0.02 (-0.36, 0.39) 0.93	0.12 (-0.21, 0.45) 0.47
	TRACP.5B	0.33 (-0.46, 1.11) 0.42	-0.12 (-0.48, 0.23) 0.50	-0.01 (-0.34, 0.32) 0.96
	25-OHD	-0.31 (-0.54, -0.09) 0.01	-0.02 (-0.16, 0.13) 0.84	-0.01 (-0.22, 0.02) 0.11
Adjust II	Without-hypertension	Reference	Reference	Reference
	With-hypertension			
	OC	0.09 (-0.22, 0.39) 0.57	-0.20 (-0.37, -0.03) 0.02	-0.11 (-0.26, 0.03) 0.13
	B-ALP	0.28 (-0.35, 0.92) 0.39	0.09 (-0.31, 0.50) 0.65	0.18 (-0.16, 0.52) 0.30
	TRACP.5B	0.51 (-0.42, 1.44) 0.29	-0.13 (-0.54, 0.27) 0.53	0.03 (-0.33, 0.39) 0.87
	25-OHD	-0.34 (-0.58, -0.10) 0.01	0.03 (-0.13, 0.19) 0.72	-0.08 (-0.21, 0.05) 0.23

In this adjusted I model, the level of B-ALP was lower by 0.46 s, 0.02 units, and 0.12 units for hypertensive males, hypertensive females, and total subjects, respectively compared with the non-hypertensive males. In adjusted II model, the level of B-ALP was higher by 0.28 units, 0.09 units, and 0.19 units for hypertensive male, hypertensive females, and total subjects, respectively, compared with the non-hypertensive group. There was a consistent correlation between hypertension and B-ALP level in males, females, and total subject. However, all the correlations had no statistical significance.

In adjusted I model, the levels of TRACP.5B was higher by 0.33 units, 0.12 units, and 0.01 units for hypertensive males, hypertensive females, and total subjects, respectively, compared with the non-hypertensive group. The level of TRACP.5B was higher by 0.51 Unit for hypertensive males and 0.03 Unit for the total subjects in adjusted II model compared with the non-hypertensive. The level of TRACP.5B was lower by 0.13 Unit for hypertensive female as well compared to the non-hypertensive. All the correlations have no statistical significance.

The level of CT was lower by 0.31 unit for hypertensive males (95% CI: -0.54 to -0.09, *P* = .01), and 0.02 unit for hypertensive males (95% CI: -0.16 to -0.13, *P* = .84) in adjusted I model compared with the non-hypertensive. Similarly in adjusted II model, there was also a consistent correlation between hypertension and 25-OHD in males (β= -0.34, 95% CI: -0.58 to 0.10, *P* = .01) and in total subjects (β= -0.08, 95% CI: -0.21 to 0.05, *P* = .23). At the same time, the level of CT was higher by 0.03 unit for hypertensive females in adjusted II model (95% CI: -0.13 to 0.19, *P* = .72) compared with non-hypertensives.

## 4 Discussion

The relationship between high blood pressure and osteoporosis has been well demonstrated. Osteoporosis and hypertension have similar causes, which involved low calcium intake, lack of vitamin D, lack of vitamin K, and high sodium intake. Epidemiological researches have shown that low vitamin D concentration is associated with high prevalence of hypertension and 25-OHD plasma concentration is also negatively correlated with diastolic blood pressure.^[15]^ Vitamin D may also affect blood pressure through multiple mechanisms. Vitamin D stimulates vascular smooth muscle cells to synthesize prostacyclin and promotes a strong vasodilation effect.^[17]^ Studies have also shown that vitamin D inhibits the proliferation of vascular smooth muscle cells, vascular calcification, and the renin-angiotensin system.^[17,18]^ Also, patient with hypertension have decreased intestinal calcium absorption and increased urinary calcium excretion which result in stimulation in PTH expression and increased skeletal calcium mobilization.^[19]^ These effects have a significant negative influence on the balance of bone reconstruction process and bone mass. In addition to the direct effects on calcium, Vitamin D can also directly stimulate osteoblasts to synthesize osteocalcin and reduce collagen synthesis as well as indirectly stimulating the activity and maturation of osteoclasts. With these effects, Vitamin D can affect the bone formation and bone resorption. Furthermore, PTH and angiotensin II receptors have been found on osteoblast cell lines that directly regulate bone turnover.^[9,10]^ Secondary PTH increase can also directly lead to increased bone resorption and high bone turnover, which may lead to bone loss.^[20]^ In our study, after the potential confounders were adjusted, 25-OHD level was found to be significantly lower in hypertensive male group than that in male without hypertension group. However, 25-OHD level in hypertensive male group was not found to be significantly lower than that in female hypertensive group. Moreover, OC level was significantly lower in hypertensive female group than that in the female without hypertension group. However, other bone metabolisms were not found to be significantly different between hypertension group and non-hypertension group. Speculation came from our team that hypertension may have negative effects on bone metabolism through the factor the low 25-OHD level in addition to other unknown factors. It is noteworthy to mention that there were no serum PTH, vitamin K and sodium intake data, so their roles in osteoporotic patients with hypertension cannot be estimated.

Serum biochemical indicators are commonly used to evaluate bone metabolism. Detection of serum bone turnover markers plays an important role in the typing, prevention, and treatment of osteoporosis. OC is an active polypeptide secreted and synthesized by osteoblasts and the level of OC reflect the function and activity of osteoblasts. Although no significant difference was found in relationship between B-ALP and hypertension, the level of OC in postmenopausal women with osteoporosis and hypertension was lower than that in osteoporotic patients without hypertension. The level of OC indicated that hypertension can lead to lower bone formation. However, the relationship between serum OC and hypertension is currently still under debate. Bezerra et al^[11]^ had studied the differences of serum OC levels in patients with metabolic syndrome. In their study, the hypertensive group had lower level of OC than the non-hypertensive group.^[21]^ In a cross-sectional study that was conducted among 162 subjects, Bezerra et al^ [11]^ show that the concentration of OC was significantly lower with hypertension than those without hypertension.^[22]^ In addition, there was an observational study of 2241 Chinese people in which the hypertensive male group had a lower serum OC level compared with those in the non-hypertensive group, but no difference was found between the female hypertensive group and female non-hypertensive group.^[23]^ To further elucidate the role of hypertension in osteoporosis, more high-quality studies are needed to explore the relationship between blood pressure and serum OC level. Although the mechanism of hypertension-related osteoporosis is still unclear, the benefits of angiotensin-converting enzyme inhibitors (ACEI) and angiotensin-converting enzyme inhibitors (ARB) in increasing bone mass and reducing fracture risk have been reported.^[24,25]^ Furthermore, angiotensin II receptor have been found expressed in osteoblasts,^[26]^ which indicate that renin-angiotensin system is directly regulating the osteoblast activity and it is one of the vital mechanisms in hypertension-related osteoporosis. Angiotensin II can stimulate the increase of intracellular Cyclic adenosine monophosphate (cAMP),^[27]^ which change the expression of Core-binding factor alpha1 (Cbfa1).^[28–30]^ The cAMP-Cbfa1 signaling mediated the osteoblast differentiation, which is further backed by the data of elevated levels of cAMP in plasma and urine of patients with osteoporosis and hypertension.^[31,32]^ More importantly, Angiotensin II can also inhibit the OC and ALP, which is regulated by the Cbfa1 promoter.^[33,34]^ However, in this study, B-ALP level was not found to be lower in hypertensive patients and this may be because ALP and BALP are not exactly the same molecules.

The most important osteoclast differentiation regulator is receptor activator of nuclear factor kappa-B ligand (RANKL), which is also mediated by cAMP.^[35]^ However, in contrast to Cbfa1, the expression of RANKL was significantly enhanced by angiotensin II.^[36]^ In addition, high blood pressure related risk factors are also conducive to promoting the expression of RANKL and osteoclast activity.^[37–41]^ It was hypothesized from our team that angiotensin II changed the expression ratio of Cbfa1/RANKL through the cAMP signaling pathway, which ultimately lead to an imbalance of bone turnover. Also, TRACP.5B is mainly derived from osteoclasts which has a strong correlation with the activity of osteoclast. The result of our study showed that TRACP.5B level was positively associated with hypertension in univariate or multivariate analysis, but there was no significant difference. Patients who have metabolic syndrome including type 2 diabetes and Paget disease were shown to have higher serum TRACP.5B level, which reflects higher bone resorption. Furthermore, hypertension was found to have no impact on bone resorption, but it requires further investigation.

The case study had several limitations. First, the case study was a retrospective cross-sectional study, and the results can only illustrate the correlation between hypertension and bone metabolism indicators, but cannot demonstrate the causal relationship between them. Second, the basic information of some patients has not been recorded in detail, which may bring some bias to the statistical results. Finally, the study investigated a relatively small number of patients in a hospital. In the future, large-scale samples and better-quality studies are needed to validate and explain the case findings.

## 5 Conclusion

The case study reveals that hypertension in newly diagnosed osteoporosis patients is negatively correlated with bone formation. This correlation can suggest that hypertension may lead to low bone turnover which may be one of the mechanisms of hypertension-induced osteoporosis.

## Author contributions

Zhuoqing Hu and Kevin Yang made substantial contributions to the conception and design, analyzed and interpreted the data, performed the literature search, and was the major contributor in the writing of the manuscript. Hao Wei, Zheng Tang, Zhihui Hu, and Baitong Chen made substantial contributions to the conception and design and revised the draft critically for important intellectual content. Chengbiao Su and Jinrong Xu made substantial contributions to the conception and design, analyzed and interpreted the data, and revised the draft critically for important intellectual content. All authors read and approved the final manuscript.

**Conceptualization:** Zhuoqing Hu, Chengbiao Su, De Cai, Jinrong Xu.

**Data curation:** Zhuoqing Hu, Kevin Yang, Chengbiao Su, De Cai, Jinrong Xu.

**Formal analysis:** Kevin Yang, Chengbiao Su, De Cai, Jinrong Xu.

**Funding acquisition:** Kevin Yang.

**Investigation:** Miaosheng Li, Baitong Chen.

**Project administration:** Zhuoqing Hu, Kevin Yang.

**Resources:** Zhihui Hu, Miaosheng Li, Baitong Chen.

**Software:** Zhihui Hu, Miaosheng Li.

**Supervision:** Hao Wei, Zheng Tang.

**Visualization:** Zhihui Hu, Hao Wei, Zheng Tang, Baitong Chen.

**Writing – original draft:** Zhihui Hu, Zheng Tang, De Cai, Jinrong Xu.

**Writing – review & editing:** Zhuoqing Hu, Kevin Yang, De Cai, Jinrong Xu.
